# 2-[(4-Hydroxy­phen­yl)diazen­yl]benzoic acid–*N*,*N*′-bis­(4-pyridylmeth­yl)oxamide (2/1)

**DOI:** 10.1107/S1600536809049228

**Published:** 2009-11-21

**Authors:** Hadi D. Arman, Tyler Miller, Pavel Poplaukhin, Edward R. T. Tiekink

**Affiliations:** a Department of Chemistry, The University of Texas at San Antonio, One UTSA Circle, San Antonio, Texas 78249-0698, USA; b Chemical Abstracts Service, 2540 Olentangy River Rd, Columbus, Ohio 43202, USA; c Department of Chemistry, University of Malaya, 50603 Kuala Lumpur, Malaysia

## Abstract

The asymmetric unit of the title co-crystal, 2C_13_H_10_N_2_O_3_·C_14_H_14_N_4_O_2_, comprises one mol­ecule of 2-(4-hydroxy­phenyl­diazen­yl)benzoic acid and half of an *N*,*N′*-bis­(4-pyridylmeth­yl)oxamide mol­ecule as the latter is disposed about an inversion centre. The most notable feature of the crystal structure is the formation of supra­molecular chains arising from hydr­oxy–pyridine O—H⋯N contacts and amide–hydr­oxy C—H⋯O contacts. These give rise to 40-membered {⋯OH⋯NNC_4_OH⋯NC_4_NC_2_NH}_2_ synthons, generating supra­molecular chains along [01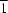]. The chains are connected into a two-dimensional array *via* C—H⋯π inter­actions. Layers, with a step-ladder topology, are consolidated into the crystal structure *via* further C—H⋯π inter­actions.

## Related literature

For background to the co-crystallization of active pharmaceutical agents and a discussion on the definition of a co-crystal, see: Shan & Zaworotko (2008 ▶); Zukerman-Schpector & Tiekink (2008 ▶). For hydrogen-bonding considerations, see: Etter (1990 ▶). For related studies on co-crystal formation, see: Broker & Tiekink (2007 ▶); Broker *et al.* (2008 ▶); Ellis *et al.* (2009 ▶). For a related salt with 2-(4-hydroxy­phenyl­diazen­yl)benzoic acid, see: Corlette & Tiekink (2009 ▶). For related structures, see: Lee & Wang (2007 ▶); Qian & Huang (2005 ▶). For co-crystals of *N*,*N′*-bis­(4-pyridylmeth­yl)oxamide, see: Wilhelm *et al.* (2008 ▶).
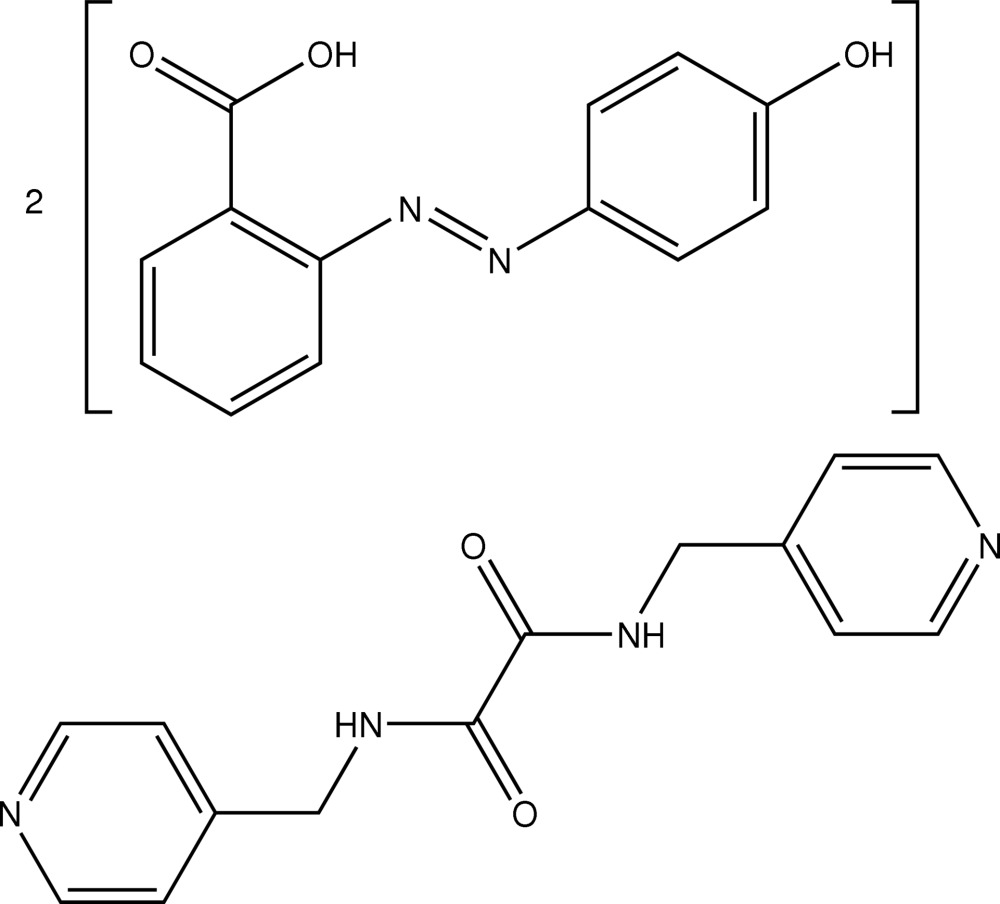


## Experimental

### 

#### Crystal data

2C_13_H_10_N_2_O_3_·C_14_H_14_N_4_O_2_
*M* *_r_* = 754.75
Triclinic, 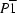

*a* = 5.523 (3) Å
*b* = 11.132 (4) Å
*c* = 15.066 (7) Å
α = 72.748 (16)°
β = 88.92 (2)°
γ = 79.43 (2)°
*V* = 869.0 (7) Å^3^
*Z* = 1
Mo *K*α radiation
μ = 0.10 mm^−1^
*T* = 98 K
0.55 × 0.31 × 0.20 mm

#### Data collection

Rigaku AFC12K/SATURN724 diffractometer
Absorption correction: multi-scan (*ABSCOR*; Higashi, 1995 ▶) *T*_min_ = 0.821, *T*_max_ = 1
6882 measured reflections
3945 independent reflections
3477 reflections with *I* > 2σ(*I*)
*R*_int_ = 0.033

#### Refinement

*R*[*F* ^2^ > 2σ(*F* ^2^)] = 0.054
*wR*(*F* ^2^) = 0.143
*S* = 1.08
3945 reflections
262 parameters
3 restraints
H-atom parameters constrained
Δρ_max_ = 0.32 e Å^−3^
Δρ_min_ = −0.28 e Å^−3^

### 

Data collection: *CrystalClear* (Rigaku/MSC, 2005 ▶); cell refinement: *CrystalClear*; data reduction: *CrystalClear*; program(s) used to solve structure: *SHELXS97* (Sheldrick, 2008 ▶); program(s) used to refine structure: *SHELXL97* (Sheldrick, 2008 ▶); molecular graphics: *ORTEPII* (Johnson, 1976 ▶) and *DIAMOND* Brandenburg, 2006 ▶); software used to prepare material for publication: *publCIF* (Westrip, 2009 ▶).

## Supplementary Material

Crystal structure: contains datablocks global, I. DOI: 10.1107/S1600536809049228/hg2597sup1.cif

Structure factors: contains datablocks I. DOI: 10.1107/S1600536809049228/hg2597Isup2.hkl

Additional supplementary materials:  crystallographic information; 3D view; checkCIF report

## Figures and Tables

**Table 1:** Hydrogen-bond geometry (Å, °)

*D*—H⋯*A*	*D*—H	H⋯*A*	*D*⋯*A*	*D*—H⋯*A*
O3—H1o⋯N3	0.84	1.79	2.568 (2)	154
N2—H1n⋯O3^i^	0.88	2.11	2.966 (2)	163
O4—H2o⋯N1^ii^	0.84	1.88	2.720 (2)	173
C5—H5⋯O2^iii^	0.95	2.34	3.187 (3)	148
C6—H6a⋯O2^i^	0.99	2.54	3.265 (3)	130
C2—H2⋯*Cg*(3)^iv^	0.95	2.76	3.542 (3)	140
C4—H4⋯*Cg*(2)	0.95	2.87	3.684 (3)	145
C11—H11⋯*Cg*(1)^v^	0.95	2.96	3.642 (3)	130

Symmetry codes: (i) 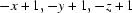; (ii) 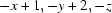; (iii) 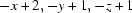; (iv) 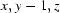; (v) 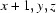. *Cg*(1), *Cg*(2) and *Cg*(3) are the centroids of the N1,C2–C5, C8–C13 and C15–C20 rings, respectively.

**Fig. 1.**
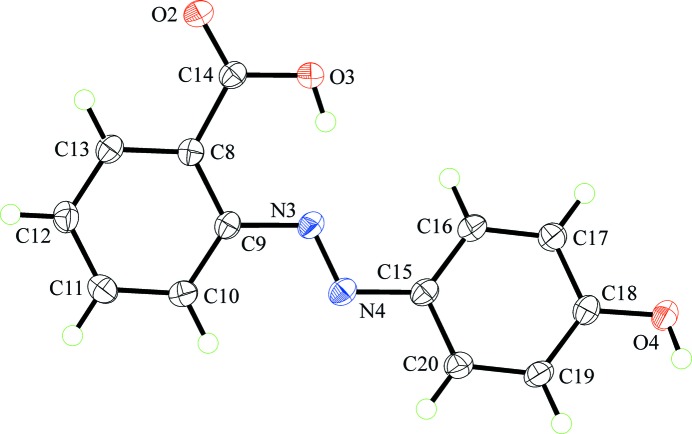
Molecular structure of 2-(4-hydroxyphenyldiazenyl)benzoic acid, one of the components of co-crystal (I), showing atom-labelling scheme and displacement ellipsoids at the 50% probability level.

**Fig. 2.**
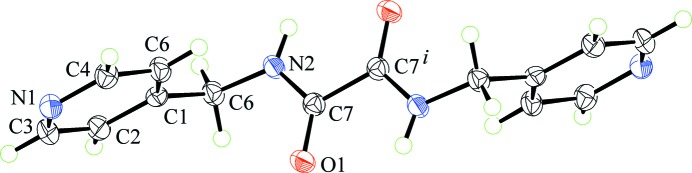
Molecular structure of N,N'-bis(4-pyridylmethyl)oxamide, one of the components of co-crystal (I), showing atom-labelling scheme and displacement ellipsoids at the 50% probability level. The molecule is located about a crystallographic centre of inversion. Symmetry operation i: 2 - x, -y, 1 - z.

**Fig. 3.**
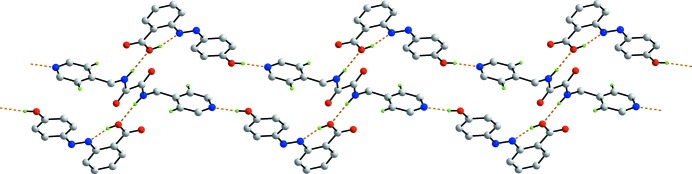
A view of a supramolecular chain mediated by O—H···N and N—H···O (orange dashed lines) hydrogen bonding showing the 40-membered {···OH···NNC4OH···NC4NC2NH}2 synthons. Colour code: S, yellow; O,red; N, blue; C, grey; and H, green.

**Fig. 4.**
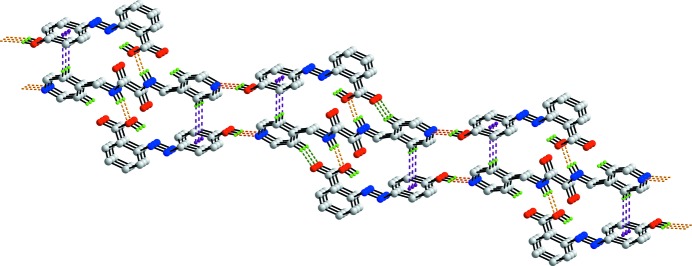
A side-on view of a layer whereby the chains shown in Figure 3 are connected to off-set chains via C—H···O (green dashed lines) and C—H···π (purple dashed lines with the ring centroid represented as a purple sphere) interactions. Colour code: S, yellow; O, red; N, blue; C, grey; and H, green.

**Fig. 5.**
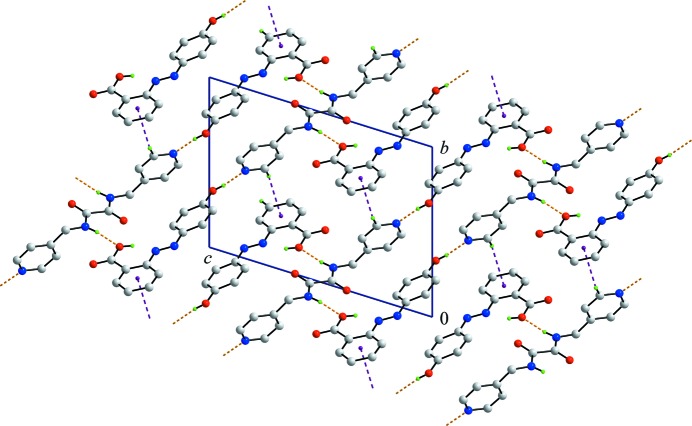
View of the stacking of layers in (I). Layers are connected by C—H···π interactions, the shortest of these is represented as purple dashed lines with each ring centroid indicated by a purple sphere. Colour code: S, yellow; O, red; N, blue; C, grey; and H, green.

## supplementary crystallographic information

### Comment

Co-crystallization of active pharmaceutical ingredients (Shan & Zaworotko, 2008) remains an active area of crystal engineering; see Zukerman-Schpector & Tiekink (2008) for terminology. As a continuation of studies into co-crystallization (Broker & Tiekink, 2007; Broker *et al.*, 2008; Ellis *et al.*, 2009), aimed at establishing a hierarchy of hydrogen bonding (Etter, 1990), the co-crystallization of 2-(4-hydroxyphenyldiazenyl)benzoic acid with *N*,*N'*-bis(4-pyridylmethyl)oxamide was investigated.

The title 2:1 co-crystal, (I), comprises a molecule of 2-(4-hydroxyphenyldiazenyl)benzoic acid (Fig. 1) and half a molecule of *N*,*N'*-bis(4-pyridylmethyl)oxamide as the latter is disposed about a centre of inversion (Fig. 2). The geometric parameters associated with the benzoic acid derivative in (I), including the intramolecular *O*—H_hydroxyl_···N_diazenyl_ hydrogen bond, Table 1, is consistent with that observed in the crystal structure of its hydrate (Qian & Huang, 2005). Similarly, the conformation of the *N*,*N'*-bis(4-pyridylmethyl)oxamide molecule is akin to those exhibited by the two independent molecules in its pure form (Lee & Wang, 2007). Unlike 2-(4-hydroxyphenyldiazenyl)benzoic (Corlette & Tiekink, 2009), the oxamide derivative has been investigated in several co-crystallization studies (*e.g.* Wilhelm *et al.*, 2008).

The primary contacts between molecules occur between the hydroxyl-O4—*H*···pyridine-N1 and N2-amide···O3-hydroxyl atoms. These combine to form 40-membered {···OH···NNC_4_OH···NC_4_NC_2_NH}_2_ synthons to generate supramolecular chains with base vector [0 1 1], Fig. 3. Chains are connected into a 2-D array *via* pyridine-C5—*H*···O2-carbonyl and pyridine-C2—H···π interactions, where the π-system is the (C15—C20 ring); Table 1 and Fig. 4. Layers, with a step-ladder topology, are consolidated into the crystal structure *via* further C—H···π interactions, Table 1 and Fig. 5.

### Experimental

Red crystals of (I) were isolated from the co-crystallization of 1:1 molar equivalents of 2-(4-hydroxyphenyldiazenyl)benzoic and *N*,*N'*-bis(4-pyridylmethyl)oxamide in an ethanol/chloroform mixture (1/1 *v*/*v*).

### Refinement

C-bound H-atoms were placed in calculated positions (C–H 0.95–0.99 Å) and were included in the refinement in the riding model approximation with *U*_iso_(H) set to 1.2*U*_eq_(C). The O– and N– bound H-atoms were located in a difference Fourier map and refined with O–H and N—H restraints of 0.840±0.001 Å and 0.88±0.001, respectively, and with *U*_iso_(H) = n*U*_eq_(carrier atom); n = 1.5 for carrier atom = O, and 1.2 for N.

### Crystal data

**Table:**

2C_13_H_10_N_2_O_3_·C_14_H_14_N_4_O_2_	*Z* = 1
*M**_r_* = 754.75	*F*(000) = 394
Triclinic, *P*1	*D*_x_ = 1.442 Mg m^−^^3^
Hall symbol: -P 1	Mo *K*α radiation, λ = 0.71069 Å
*a* = 5.523 (3) Å	Cell parameters from 3695 reflections
*b* = 11.132 (4) Å	θ = 2.0–40.6°
*c* = 15.066 (7) Å	µ = 0.10 mm^−^^1^
α = 72.748 (16)°	*T* = 98 K
β = 88.92 (2)°	Block, red
γ = 79.43 (2)°	0.55 × 0.31 × 0.20 mm
*V* = 869.0 (7) Å^3^	

### Data collection

**Table:**

Rigaku AFC12K/SATURN724 diffractometer	3945 independent reflections
Radiation source: fine-focus sealed tube	3477 reflections with *I* > 2σ(*I*)
graphite	*R*_int_ = 0.033
ω scans	θ_max_ = 27.5°, θ_min_ = 2.0°
Absorption correction: multi-scan (*ABSCOR*; Higashi, 1995)	*h* = −6→7
*T*_min_ = 0.821, *T*_max_ = 1	*k* = −14→14
6882 measured reflections	*l* = −19→19

### Refinement

**Table:**

Refinement on *F*^2^	Primary atom site location: structure-invariant direct methods
Least-squares matrix: full	Secondary atom site location: difference Fourier map
*R*[*F*^2^ > 2σ(*F*^2^)] = 0.054	Hydrogen site location: inferred from neighbouring sites
*wR*(*F*^2^) = 0.143	H-atom parameters constrained
*S* = 1.08	*w* = 1/[σ^2^(*F*_o_^2^) + (0.0675*P*)^2^ + 0.3418*P*] where *P* = (*F*_o_^2^ + 2*F*_c_^2^)/3
3945 reflections	(Δ/σ)_max_ < 0.001
262 parameters	Δρ_max_ = 0.32 e Å^−^^3^
3 restraints	Δρ_min_ = −0.28 e Å^−^^3^

### Special details

**Table:**

Geometry. All e.s.d.'s (except the e.s.d. in the dihedral angle between two l.s. planes) are estimated using the full covariance matrix. The cell e.s.d.'s are taken into account individually in the estimation of e.s.d.'s in distances, angles and torsion angles; correlations between e.s.d.'s in cell parameters are only used when they are defined by crystal symmetry. An approximate (isotropic) treatment of cell e.s.d.'s is used for estimating e.s.d.'s involving l.s. planes.
Refinement. Refinement of *F*^2^ against ALL reflections. The weighted *R*-factor *wR* and goodness of fit *S* are based on *F*^2^, conventional *R*-factors *R* are based on *F*, with *F* set to zero for negative *F*^2^. The threshold expression of *F*^2^ > σ(*F*^2^) is used only for calculating *R*-factors(gt) *etc*. and is not relevant to the choice of reflections for refinement. *R*-factors based on *F*^2^ are statistically about twice as large as those based on *F*, and *R*- factors based on ALL data will be even larger.

### Fractional atomic coordinates and isotropic or equivalent isotropic displacement parameters (Å^2^)

**Table:**

	*x*	*y*	*z*	*U*_iso_*/*U*_eq_	
O1	1.0476 (2)	−0.00128 (11)	0.38418 (8)	0.0287 (3)	
O2	0.7677 (2)	0.68650 (12)	0.51985 (8)	0.0293 (3)	
O3	0.5687 (2)	0.83724 (11)	0.40195 (8)	0.0264 (3)	
H1O	0.5873	0.8673	0.3446	0.040*	
O4	0.0094 (2)	1.33786 (11)	−0.01572 (8)	0.0298 (3)	
H2O	0.0524	1.3892	−0.0638	0.045*	
N1	0.8421 (3)	0.48340 (13)	0.16157 (9)	0.0268 (3)	
N2	0.7290 (2)	0.10782 (13)	0.44396 (9)	0.0238 (3)	
H1N	0.6700	0.1179	0.4965	0.029*	
N3	0.7538 (2)	0.87835 (12)	0.24039 (9)	0.0222 (3)	
N4	0.7707 (2)	0.94258 (12)	0.15667 (9)	0.0230 (3)	
C1	0.6793 (3)	0.27688 (14)	0.28993 (10)	0.0216 (3)	
C2	0.5771 (3)	0.32819 (15)	0.19998 (11)	0.0267 (3)	
H2	0.4492	0.2940	0.1805	0.032*	
C3	0.6641 (3)	0.42965 (16)	0.13916 (11)	0.0299 (4)	
H3	0.5930	0.4631	0.0778	0.036*	
C4	0.9377 (3)	0.43484 (15)	0.24874 (11)	0.0264 (3)	
H4	1.0628	0.4723	0.2666	0.032*	
C5	0.8634 (3)	0.33260 (15)	0.31435 (11)	0.0252 (3)	
H5	0.9376	0.3011	0.3752	0.030*	
C6	0.5892 (3)	0.16366 (15)	0.35621 (11)	0.0244 (3)	
H6A	0.4150	0.1914	0.3693	0.029*	
H6B	0.5944	0.0966	0.3250	0.029*	
C7	0.9439 (3)	0.02757 (14)	0.44999 (10)	0.0221 (3)	
C8	0.9611 (3)	0.71595 (14)	0.37441 (10)	0.0208 (3)	
C9	0.9623 (3)	0.78081 (14)	0.27915 (10)	0.0212 (3)	
C10	1.1625 (3)	0.74920 (15)	0.22689 (11)	0.0258 (3)	
H10	1.1636	0.7929	0.1624	0.031*	
C11	1.3589 (3)	0.65398 (16)	0.26952 (12)	0.0279 (4)	
H11	1.4954	0.6330	0.2341	0.034*	
C12	1.3583 (3)	0.58874 (15)	0.36356 (12)	0.0265 (3)	
H12	1.4932	0.5229	0.3922	0.032*	
C13	1.1604 (3)	0.61987 (15)	0.41561 (11)	0.0237 (3)	
H13	1.1606	0.5753	0.4800	0.028*	
C14	0.7599 (3)	0.74462 (14)	0.43795 (10)	0.0222 (3)	
C15	0.5672 (3)	1.03918 (14)	0.11805 (10)	0.0224 (3)	
C16	0.3394 (3)	1.05976 (15)	0.15929 (11)	0.0233 (3)	
H16	0.3115	1.0047	0.2186	0.028*	
C17	0.1561 (3)	1.16005 (15)	0.11346 (11)	0.0251 (3)	
H17	0.0018	1.1737	0.1414	0.030*	
C18	0.1956 (3)	1.24225 (15)	0.02580 (11)	0.0238 (3)	
C19	0.4230 (3)	1.22184 (16)	−0.01495 (11)	0.0263 (3)	
H19	0.4520	1.2777	−0.0738	0.032*	
C20	0.6053 (3)	1.12046 (15)	0.03040 (11)	0.0252 (3)	
H20	0.7582	1.1057	0.0018	0.030*	

### Atomic displacement parameters (Å^2^)

**Table:**

	*U*^11^	*U*^22^	*U*^33^	*U*^12^	*U*^13^	*U*^23^
O1	0.0295 (6)	0.0338 (6)	0.0211 (6)	0.0007 (5)	0.0015 (5)	−0.0098 (5)
O2	0.0308 (6)	0.0344 (6)	0.0189 (6)	−0.0012 (5)	0.0003 (5)	−0.0055 (5)
O3	0.0263 (6)	0.0291 (6)	0.0201 (5)	0.0010 (5)	0.0023 (4)	−0.0057 (5)
O4	0.0258 (6)	0.0295 (6)	0.0262 (6)	−0.0009 (5)	−0.0010 (5)	0.0011 (5)
N1	0.0282 (7)	0.0257 (6)	0.0233 (7)	−0.0017 (5)	0.0007 (5)	−0.0045 (5)
N2	0.0236 (7)	0.0273 (6)	0.0176 (6)	−0.0007 (5)	0.0000 (5)	−0.0048 (5)
N3	0.0253 (7)	0.0217 (6)	0.0183 (6)	−0.0034 (5)	−0.0022 (5)	−0.0044 (5)
N4	0.0268 (7)	0.0230 (6)	0.0194 (6)	−0.0057 (5)	−0.0020 (5)	−0.0059 (5)
C1	0.0209 (7)	0.0220 (7)	0.0210 (7)	0.0009 (5)	0.0006 (6)	−0.0080 (6)
C2	0.0292 (8)	0.0264 (7)	0.0245 (8)	−0.0034 (6)	−0.0056 (6)	−0.0085 (6)
C3	0.0388 (9)	0.0290 (8)	0.0190 (7)	−0.0020 (7)	−0.0059 (6)	−0.0049 (6)
C4	0.0223 (7)	0.0269 (7)	0.0280 (8)	−0.0019 (6)	−0.0033 (6)	−0.0066 (6)
C5	0.0237 (8)	0.0273 (7)	0.0212 (7)	−0.0008 (6)	−0.0038 (6)	−0.0045 (6)
C6	0.0237 (7)	0.0269 (7)	0.0207 (7)	−0.0029 (6)	−0.0025 (6)	−0.0053 (6)
C7	0.0233 (7)	0.0217 (7)	0.0212 (8)	−0.0055 (6)	0.0013 (6)	−0.0054 (6)
C8	0.0211 (7)	0.0209 (7)	0.0211 (7)	−0.0044 (5)	−0.0010 (6)	−0.0071 (6)
C9	0.0226 (7)	0.0206 (7)	0.0204 (7)	−0.0044 (6)	−0.0025 (6)	−0.0059 (6)
C10	0.0289 (8)	0.0261 (7)	0.0205 (7)	−0.0036 (6)	0.0026 (6)	−0.0054 (6)
C11	0.0249 (8)	0.0308 (8)	0.0290 (8)	−0.0035 (6)	0.0046 (6)	−0.0114 (7)
C12	0.0231 (8)	0.0269 (7)	0.0277 (8)	0.0003 (6)	−0.0043 (6)	−0.0079 (6)
C13	0.0249 (8)	0.0254 (7)	0.0204 (7)	−0.0045 (6)	−0.0031 (6)	−0.0064 (6)
C14	0.0252 (8)	0.0222 (7)	0.0198 (7)	−0.0052 (6)	−0.0013 (6)	−0.0067 (6)
C15	0.0267 (8)	0.0228 (7)	0.0186 (7)	−0.0059 (6)	−0.0023 (6)	−0.0063 (6)
C16	0.0253 (8)	0.0247 (7)	0.0197 (7)	−0.0073 (6)	−0.0008 (6)	−0.0045 (6)
C17	0.0222 (7)	0.0274 (7)	0.0248 (8)	−0.0061 (6)	0.0004 (6)	−0.0055 (6)
C18	0.0246 (8)	0.0238 (7)	0.0226 (7)	−0.0045 (6)	−0.0042 (6)	−0.0059 (6)
C19	0.0290 (8)	0.0281 (8)	0.0186 (7)	−0.0058 (6)	0.0002 (6)	−0.0016 (6)
C20	0.0256 (8)	0.0290 (8)	0.0201 (7)	−0.0046 (6)	0.0003 (6)	−0.0063 (6)

### Geometric parameters (Å, °)

**Table:**

O1—C7	1.2304 (19)	C6—H6B	0.9900
O2—C14	1.2088 (19)	C7—C7^i^	1.542 (3)
O3—C14	1.3276 (19)	C8—C13	1.393 (2)
O3—H1O	0.8402	C8—C9	1.403 (2)
O4—C18	1.3450 (19)	C8—C14	1.505 (2)
O4—H2O	0.8401	C9—C10	1.398 (2)
N1—C3	1.337 (2)	C10—C11	1.383 (2)
N1—C4	1.340 (2)	C10—H10	0.9500
N2—C7	1.335 (2)	C11—C12	1.387 (2)
N2—C6	1.451 (2)	C11—H11	0.9500
N2—H1N	0.8801	C12—C13	1.386 (2)
N3—N4	1.2632 (19)	C12—H12	0.9500
N3—C9	1.427 (2)	C13—H13	0.9500
N4—C15	1.403 (2)	C15—C20	1.399 (2)
C1—C2	1.390 (2)	C15—C16	1.404 (2)
C1—C5	1.391 (2)	C16—C17	1.379 (2)
C1—C6	1.516 (2)	C16—H16	0.9500
C2—C3	1.384 (2)	C17—C18	1.405 (2)
C2—H2	0.9500	C17—H17	0.9500
C3—H3	0.9500	C18—C19	1.398 (2)
C4—C5	1.389 (2)	C19—C20	1.381 (2)
C4—H4	0.9500	C19—H19	0.9500
C5—H5	0.9500	C20—H20	0.9500
C6—H6A	0.9900		
			
C14—O3—H1O	108.7	C10—C9—N3	123.07 (14)
C18—O4—H2O	112.5	C8—C9—N3	116.96 (13)
C3—N1—C4	116.75 (14)	C11—C10—C9	119.74 (15)
C7—N2—C6	120.86 (13)	C11—C10—H10	120.1
C7—N2—H1N	116.6	C9—C10—H10	120.1
C6—N2—H1N	122.1	C10—C11—C12	120.63 (15)
N4—N3—C9	115.26 (13)	C10—C11—H11	119.7
N3—N4—C15	115.72 (13)	C12—C11—H11	119.7
C2—C1—C5	117.49 (14)	C13—C12—C11	119.81 (15)
C2—C1—C6	119.45 (14)	C13—C12—H12	120.1
C5—C1—C6	123.05 (14)	C11—C12—H12	120.1
C3—C2—C1	119.17 (16)	C12—C13—C8	120.65 (15)
C3—C2—H2	120.4	C12—C13—H13	119.7
C1—C2—H2	120.4	C8—C13—H13	119.7
N1—C3—C2	123.90 (15)	O2—C14—O3	119.58 (14)
N1—C3—H3	118.0	O2—C14—C8	122.19 (14)
C2—C3—H3	118.0	O3—C14—C8	118.23 (13)
N1—C4—C5	123.41 (16)	C20—C15—N4	114.33 (14)
N1—C4—H4	118.3	C20—C15—C16	119.49 (14)
C5—C4—H4	118.3	N4—C15—C16	126.18 (14)
C4—C5—C1	119.28 (15)	C17—C16—C15	119.85 (15)
C4—C5—H5	120.4	C17—C16—H16	120.1
C1—C5—H5	120.4	C15—C16—H16	120.1
N2—C6—C1	114.78 (14)	C16—C17—C18	120.54 (15)
N2—C6—H6A	108.6	C16—C17—H17	119.7
C1—C6—H6A	108.6	C18—C17—H17	119.7
N2—C6—H6B	108.6	O4—C18—C19	122.69 (14)
C1—C6—H6B	108.6	O4—C18—C17	117.82 (14)
H6A—C6—H6B	107.5	C19—C18—C17	119.49 (14)
O1—C7—N2	125.14 (15)	C20—C19—C18	119.95 (15)
O1—C7—C7^i^	121.86 (17)	C20—C19—H19	120.0
N2—C7—C7^i^	112.99 (16)	C18—C19—H19	120.0
C13—C8—C9	119.19 (14)	C19—C20—C15	120.66 (15)
C13—C8—C14	116.22 (14)	C19—C20—H20	119.7
C9—C8—C14	124.57 (14)	C15—C20—H20	119.7
C10—C9—C8	119.98 (14)		
			
C9—N3—N4—C15	−179.67 (12)	C9—C10—C11—C12	0.5 (3)
C5—C1—C2—C3	0.9 (2)	C10—C11—C12—C13	−0.6 (3)
C6—C1—C2—C3	−178.53 (14)	C11—C12—C13—C8	0.2 (2)
C4—N1—C3—C2	−0.5 (2)	C9—C8—C13—C12	0.3 (2)
C1—C2—C3—N1	−0.5 (3)	C14—C8—C13—C12	−178.21 (14)
C3—N1—C4—C5	0.9 (2)	C13—C8—C14—O2	−1.2 (2)
N1—C4—C5—C1	−0.5 (2)	C9—C8—C14—O2	−179.56 (15)
C2—C1—C5—C4	−0.5 (2)	C13—C8—C14—O3	178.70 (14)
C6—C1—C5—C4	178.93 (14)	C9—C8—C14—O3	0.3 (2)
C7—N2—C6—C1	−79.82 (19)	N3—N4—C15—C20	171.10 (13)
C2—C1—C6—N2	172.60 (14)	N3—N4—C15—C16	−8.5 (2)
C5—C1—C6—N2	−6.8 (2)	C20—C15—C16—C17	−0.4 (2)
C6—N2—C7—O1	3.7 (2)	N4—C15—C16—C17	179.19 (15)
C6—N2—C7—C7^i^	−176.57 (15)	C15—C16—C17—C18	−0.2 (2)
C13—C8—C9—C10	−0.4 (2)	C16—C17—C18—O4	179.60 (14)
C14—C8—C9—C10	178.01 (14)	C16—C17—C18—C19	0.0 (2)
C13—C8—C9—N3	180.00 (13)	O4—C18—C19—C20	−178.74 (15)
C14—C8—C9—N3	−1.6 (2)	C17—C18—C19—C20	0.9 (2)
N4—N3—C9—C10	−5.8 (2)	C18—C19—C20—C15	−1.5 (2)
N4—N3—C9—C8	173.84 (13)	N4—C15—C20—C19	−178.39 (14)
C8—C9—C10—C11	0.0 (2)	C16—C15—C20—C19	1.2 (2)
N3—C9—C10—C11	179.58 (15)		

Symmetry codes: (i) −*x*+2, −*y*, −*z*+1.

### Hydrogen-bond geometry (Å, °)

**Table:**

*D*—H···*A*	*D*—H	H···*A*	*D*···*A*	*D*—H···*A*
O3—H1o···N3	0.84	1.79	2.568 (2)	154
N2—H1n···O3^ii^	0.88	2.11	2.966 (2)	163
O4—H2o···N1^iii^	0.84	1.88	2.720 (2)	173
C5—H5···O2^iv^	0.95	2.34	3.187 (3)	148
C6—H6a···O2^ii^	0.99	2.54	3.265 (3)	130
C2—H2···Cg(3)^v^	0.95	2.76	3.542 (3)	140
C4—H4···Cg(2)	0.95	2.87	3.684 (3)	145
C11—H11···Cg(1)^vi^	0.95	2.96	3.642 (3)	130

Symmetry codes: (ii) −*x*+1, −*y*+1, −*z*+1; (iii) −*x*+1, −*y*+2, −*z*; (iv) −*x*+2, −*y*+1, −*z*+1; (v) *x*, *y*−1, *z*; (vi) *x*+1, *y*, *z*.
